# Diagnostic Yield of Next Generation Sequencing in Genetically Undiagnosed Patients with Primary Immunodeficiencies: a Systematic Review

**DOI:** 10.1007/s10875-019-00656-x

**Published:** 2019-06-28

**Authors:** Hemmo A. F. Yska, Kim Elsink, Taco W. Kuijpers, Geert W. J. Frederix, Mariëlle E. van Gijn, Joris M. van Montfrans

**Affiliations:** 1 Department of Pediatric Immunology and Infectious Diseases, University Medical Centre Utrecht, Utrecht, The Netherlands; 2 Department of Pediatric Hematology, Immunology and Infectious Diseases, Emma Children’s Hospital, Academic Medical Centre, University of Amsterdam, Amsterdam, The Netherlands; 3 Department of Medical Genetics, University Medical Centre Utrecht, Utrecht, The Netherlands; 4 Julius Center for Health Sciences and Primary Care, University Medical Centre Utrecht, Utrecht, The Netherlands

**Keywords:** Primary immunodeficiency (PID), next generation sequencing (NGS), diagnostic yield, technological performance, clinical performance, whole exome sequencing (WES), whole genome sequencing (WGS)

## Abstract

**Background:**

As the application of next generation sequencing (NGS) is moving to earlier stages in the diagnostic pipeline for primary immunodeficiencies (PIDs), re-evaluation of its effectiveness is required. The aim of this study is to systematically review the diagnostic yield of NGS in PIDs.

**Methods:**

PubMed and Embase databases were searched for relevant studies. Studies were eligible when describing the use of NGS in patients that had previously been diagnosed with PID on clinical and/or laboratory findings. Relevant data on study characteristics, technological performance and diagnostic yield were extracted.

**Results:**

Fourteen studies were eligible for data extraction. Six studies described patient populations from specific PID subcategories. The remaining studies included patients with unsorted PIDs. The studies were based on populations from Italy, Iran, Turkey, Thailand, the Netherlands, Norway, Saudi Arabia, Sweden, the UK, and the USA. Eight studies used an array-based targeted gene panel, four used WES in combination with a PID filter, and two used both techniques. The mean reported reading depth ranged from 98 to 1337 times. Five studies described the sensitivity of the applied techniques, ranging from 83 to 100%, whereas specificity ranged from 45 to 99.9%. The percentage of patients who were genetically diagnosed ranged from 15 to 79%. Several studies described clinical implications of the genetic findings.

**Discussion:**

NGS has the ability to contribute significantly to the identification of molecular mechanisms in PID patients. The diagnostic yield highly depends on population and on the technical circumstances under which NGS is employed. Further research is needed to determine the exact diagnostic yield and clinical implications of NGS in patients with PID.

## Introduction

Primary immunodeficiencies (PIDs) are a diverse group of congenital diseases affecting different parts of the immune system. Patients usually present with a varying degree of recurrent, unusual or severe infections, autoimmunity, autoinflammation, allergy and/or malignancies [1–3]. Identification and clinical diagnosis of the exact type of PID have important consequences in terms of prognosis, treatment, and genetic counseling [4–7]. However, phenotypic and genotypic heterogeneity, causing atypical presentations and overlap of symptoms between diseases, impedes reaching a definitive molecular diagnosis [8–11]. The introduction of NGS-based sequencing techniques, facilitating testing of panels of disease-related genes, can overcome these diagnostic difficulties [12]. Currently, over 360 genes involved in immunodeficiencies have been identified and are classified on a yearly basis by the International Union of Immunological Societies (IUIS) [13].

Several DNA sequencing techniques are currently being used to detect disease-causing mutations. Until 2010, in the clinical situation, routine genetic analyses were primarily performed by means of Sanger sequencing [14]. The introduction of next generation sequencing (NGS) has, next to its contribution to the expansion of the list of genes known to cause PIDs, provided a much quicker and now cheaper way to evaluate large portions of the genome [9, 15, 16]. Especially in situations where there is no obvious candidate gene, NGS is preferred to Sanger sequencing [14]. However, array-based targeted gene panels and whole exome sequencing (WES) may have the disadvantage of insufficient coverage of specific regions of the genome thereby creating the possibility of missing mutations. Whole genome sequencing (WGS) provides a more complete picture, with improved identification of CNV’s and other genomic rearrangements, but complicates analysis due to the generation of even larger amounts of data [17]. Moreover, due to the frequent use of short read technologies, limitations such as GC bias, difficulties with mapping to repetitive elements, trouble discriminating paralogous sequences and identification of large indels complicate its use. In some cases, Sanger sequencing therefore remains essential in the confirmation of mutations identified by NGS [14].

Due to technical advances and the continuously decreasing costs of NGS [18], its place in the diagnostic pipeline of PID requires re-evaluation as it is moving to the earlier stages. In order to do so, this review aims to describe the technological performance and diagnostic yield of NGS in PID patients.

## Methods

### Search Strategy

We performed a systematic review in order to analyze the current literary framework describing the use of NGS in PIDs. We searched the Pubmed and Embase databases for relevant studies in June 2018 using the terms primary immunodeficiency and *PID* combined with next generation sequencing and related techniques including whole exome sequencing and whole genome sequencing. The full search string is presented in Appendix 1, Table 4. We retrieved 219 studies from Pubmed and 297 from Embase. No further relevant studies were found in the reference lists of the included studies.

### Eligibility

We aimed to study the use of NGS in a clinical setting. To this end, we selected all papers that described the use of NGS in patients that had previously been clinically diagnosed with a primary immunodeficiency or were highly suspected of having one according to clinical parameters as described by the authors. Exclusion criteria included prior knowledge of a genetic mutation, and the use of NGS for diagnosing other disease categories than PID according to the IUIS 2017 guidelines. We disregarded studies describing diseases with both PID-non-PID-related causes. We further rejected studies that included less than *n* = 10 patients, and studies with results that were not written in English. Case series of patients within the same family were also excluded due to a high probability of all patients having the same causative mutation.

### Selection of Studies

**Fig. 1:**
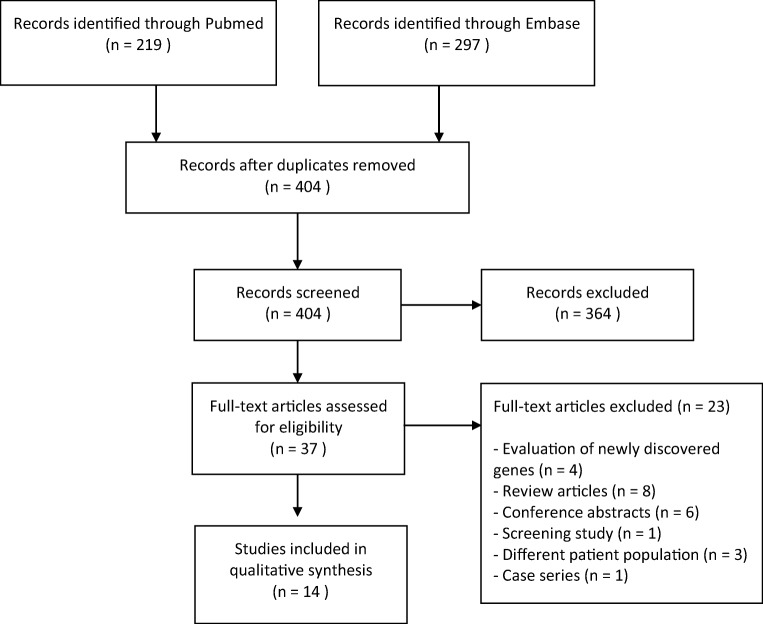
PRISMA flow diagram

After duplicate removal, all 404 remaining studies were first screened by title and abstract by a first author (HY), followed by a second author (KE). After agreeing on conflicts, 367 studies were excluded. A significant amount consisted of conference abstracts that had not yet been excluded in earlier stages. The 37 studies that remained were assessed by both authors by analysis of the full text. Fourteen studies were determined to be eligible for data extraction. The selection of studies is summarized in Fig. 1.

### Data Extraction

The following background information was extracted from the eligible papers: year of publication, country, population, number of patients included, and previous genetic analysis technique. Technical variables included the sequencing technique, sequencing platform, number of genes included and the rationale behind it, coverage of base pairs, reading depth, sensitivity, specificity, and additional analyses performed by the researchers in order to ensure completeness. Finally, information regarding the diagnostic yield, rationale behind evaluation of mutations, analyses, and clinical implications were collected.

### Critical Appraisal

Most appraisal tools for diagnostic studies were designed for studies that compare a reference standard with an index test, which was not the focus of this article. Therefore, it was decided to use a modified version of the 2015 STARD criteria [19]. Using this list, the included studies were critically assessed for possible bias and completeness of reporting. In short, articles were scored on fourteen items that mainly reflected population background, rationale behind and quality of the analysis and general completeness. If four or more of the included items were found to be missing, completeness of reporting was considered inadequate.

## Results

### Study Characteristics

The fourteen studies eligible for data extraction are described in Table 1. Eight of these used NGS in a mixed PID population; the other six studies described patients from specific subcategories of PID. The number of included patients in the studies describing unsorted populations ranged from 15 to 278 (median = 41). The six papers focusing on specific PID subcategories included 19–696 patients (median = 38). Eight studies were based on Western patient populations. Others were based on populations from Iran, Turkey, Thailand, and Saudi Arabia. Several studies did not describe clinical characteristics such as gender, specific symptoms, and severity of the phenotypes in the affected patients in detail. Of the studies that did specify these data, seven described patients who received genetic testing prior to NGS, whereas patients from several other studies (Yu et al. and Maffucci et al.) had not [20, 21].

**Table 1:** Study characteristics

Authors	Year	Country	Disease(s) included	*N*	Previous genetic work-up
Analysis of unsorted PIDs					
Nijman et al.	2014	The Netherlands	CID, ALPS, granulopenia, HLH/XLP	26	Extensive genetic testing.
Stoddard et al.	2014	USA	Unspecified	120	9 patients had received extensive testing
Moens et al.	2014	Sweden	Patients without any knowledge of the disease-causing mutation and patients with agammaglobulinemia without *BTK* mutations	15	NR
Al-Mousa	2016	Saudi Arabia	Patients suspected of having PIDs but without a confirmed genetic diagnosis^1^	139	70/139 complicated cases had received extensive genetic testing.
Gallo	2016	Italy	Patients with a clinical history highly suggestive of a primary immunological defect^2^ in combination with abnormal immune parameters^3^.	45	6 patients had undergone extensive diagnostic testing.
Stray-Pedersen	2017	USA + Norway	Broad range of phenotypes^4^	278	Conventional genetic testing.
Rae	2018	UK	Phenotype compatible with PID and a diagnosis according to the European Society for Immunodeficiencies (ESID)	27	NR
Bisgin et al.	2018	Turkey	Patients with immunodeficiency diagnosis^5^	37	None
Analysis of specific PID disease categories					
Maffucci	2016	USA	CVID^6^	50	NR
Yu	2016	USA	SCID	20	None
Mukda	2017	Thailand	HLH	25	NR
Erman	2017	Turkey	SCID	19	Several SCID genes were excluded in six patients by Sanger sequencing before participation
Abolhassani et al.	2018	Iran	Combined immunodeficiencies^7^	696^8^	NR
Abolhassani et al.	2019	Iran	Patients with primary antibody deficiencies, including CVID, agammglobulinemia, HIgM syndrome, IgA deficiency, and other (unspecified) types	545^9^	77 were diagnosed with agammaglobulinemia (*n* = 49) and HIgM (*n* = 28) using conventional genetic methods

^1^SCID, CID, chronic granulomatous disease, hyper-IgE syndrome, common variable immunodeficiency, ill-defined immunodeficiency, hyper-IgM syndrome, agammaglobulinemia, hypogammaglobulinemia, bare lymphocyte syndrome, ataxia telangiectasia like, autoimmune enteropathy, hemophagocytic lymphohistiocytosis, Omenn disease, autoimmune lymphoproliferative syndrome, autoinflammatory disease, chronic mucocutaneous candidiasis, Griscelli syndrome, lymphoproliferative disease, neutropenia, and Chediak-Higashi syndrome

^2^Opportunistic infections, granuloma, CMC, intractable diarrhea, bronchiectasis, and severe autoimmunity

^3^Abnormal lymphocyte subsets (absolute count < 2 SD of normal values according to ESID criteria); proliferative response to mitogens < 10% of the levels measured in the control subject; absent/poor specific antibody response; hypogammaglobulinemia; elevated IgE levels (> 2000 kU/l); severe impairment of cytolytic activity; and alteration of class switch recombination (CSR) with or without hyper-IgM

^4^One of ten subgroups: (I) antibody deficiency (humoral deficiency that does not fulfill the diagnostic criteria for common variable immunodeficiency (CVID)); (II) autoimmune disease; (III) autoinflammatory disorder; (IV) severe combined immunodeficiency (SCID); (V) combined immunodeficiency (not SCID) and selective T cell deficiency; (VI) CVID; (VII) defect in innate immunity, including mucocutaneous candidiasis, hyper-IgE syndrome, Mendelian susceptibility to mycobacterial disease, and complement deficiency; (VIII) lymphoproliferative disease, HLH, and natural killer cell-deficiency; (IX) neutrophil defect or congenital condition with bone marrow failure, such as dyskeratosis congenita and Fanconi-like phenotype, anemia, and thrombocytopenia; and (X) immuno-osseous dysplasia, chromosomal disorder, or other syndromal PIDD

^5^SCID, agammaglobulinemia, HIGM, HIES, Wiskott-Aldrich syndrome, MSMD, CMCD

^6^Severe phenotype in half of all cases

^7^Including SCID, Omenn phenotype, hIGM, partial T cell defects, HIES, Wiskott-Aldrich syndrome, DNA repair defect syndromes, dyskeratosis congenital, ectodermal dysplasia, and other atypical and incomplete syndromic CIDs

^8^243 enrolled for sequencing

^9^126 patients were enrolled for exome sequencing

*NR*, not reported

### Technical Performance

Of the 14 eligible studies, eight used an array-based targeted gene panel, four used WES combined with a PID filter, and two studies used both techniques. The technical evaluation of these techniques is described in Table 2. Most authors used one of three sequencing platforms: SOLiD (*n* = 1), ion system (*n* = 4), or illumina (*n* = 8), and one study did not specify the used platform. The number of genes included in the analysis ranged from 12 to 365 in the specific PID group and from 60 to 571 in the mixed population group. Not all authors clarified their choice for the selection of the included genes. Reading depth and/or coverage were presented in all papers, except in one study [22]. Complete and in-depth comparison of efficacy of detecting mutations between studies could not be performed due to incomplete presentation of sufficient technical parameters. The studies that did mention these parameters reported a minimum of 88% of base pairs covered at least once. A total of five studies performed additional copy number variant (CNV) analyses on their samples (Table 3), which increased the diagnostic yield by on average 4.2%.

**Table 2:** Technical performance of NGS-based tests

Authors	Sequencing technique	Sequencing platform	Number of genes	Source	Coverage	Reading depth	Sensitivity	Specificity	Remarks
Analysis of unsorted PIDs									
Nijman et al.	Array-based targeted PID panel	AB SOLiD 5500XL	170	2011 IUIS criteria	90.4–95.2% at 20 times	338^m^ (array enrichment strategy)	SNPs: 99.5%	SNPs: 99.9%	9 genes with low reads. Apart from these 15–20% of the genes had 1 or multiple exons with low coverage
						192^me^ (SureSelect enrichment strategy)	Indels: 85%		
Stoddard et al.	Array-based targeted PID panel	Ion PGM 200	173	“Known or highly suspected to be associated to particular PIDs.”	100% for most target genes	305^m^	98.1%	99.9%	-2 genes excluded from evaluation.
									-Problems with INDEL characterization
Moens et al.	Array-based targeted PID panel	Illumina	179	“Selected based on the 2009 IUIS PID classification + reports on additional genes presented at the 2010 ESID meeting”.	95% at least 1 × 88% at least 20 times	1304^a^	83%	89.7%	1 gene with a low read depth + several genes with low exon read depth.
Al-Mousa et al.	Array-based targeted PID panel	Ion proton instrument	162	2014 IUIS criteria	96,51% for all genes	461^a^	SNPs and short indels 96%	SNPs and short indels 88.2%	9 genes had < 90% coverage. 2 genes were concluded to have inadequate coverage.
							CNVs 100%	CNVs 45%	
Gallo et al.	Array-based targeted PID panel + WES	Array-based targeted PID panel: Illumina MiSeq	571	Based on broad searches in literature, PubMed queries and expert suggestions, including “68 genes known or predicted to be related to PID or immune regulation.”	Array-based targeted PID panel: 98.9% at least 10 times.	Array-based targeted PID panel: 580^a^	98.7%	NR	NA
		WES: Illumina HiSeq 2500			WES: 97% at least 10 times.				
Stray-Pedersen et al.	WES	Illumina HiSeq 2500	475	“A combination of known or candidate PID genes from the Resource of Asian PID disease, IUIS and supplemented with genes known to affect telomere length or cause Fanconi anemia. If no result were found, complete exome data was analyzed for possible variants.”	> 90% at least 20 times	100^a^	NR	NR	NA
Rae et al.	Array-based targeted PID panel	Illumina NexSeq 500	242	The TruSight One 4813 gene exon panel was “filtered to include only the virtual PID gene panel”.	99.5% at least 1 times	98^a^	NR	NR	NA
					96.2% at least 30 times				
Bisgin et al.	Array-based targeted multigene panel	Illumina MiSeq	60	Based on flow cytometry results, multigene panels were selected according to immunophenotype	Minimum absolute coverage of 200 times	NR	NR	NR	NA
Analysis of specific PID disease categories									
Maffucci et al.	WES	Illumina HiSeq 2500	269	“Genes associated with PIDs.”	Bp reads < 2 times were excluded	NR	NR	NR	NA
Yu et al.	Array-based targeted PID panel	Illumina HiSeq 2000	46	Selection, including all SCID-causing genes, from 191 “genes associated with various primary immunodeficiency phenotypes and 5 genes located in the 22q11.2 deletion region.”	97% at least 100 times	1337^a^	NR	NR	6 exons from 5 genes showed insufficient coverage. 15 exons contained highly homologous regions requiring Sanger confirmation.
Mukda et al.	WES	Ion proton system	12	“HLH-associated genes”.	88% at least 20 times	104.87^m^	NR	NR	NA
Erman et al.	Array-based targeted PID panel	Illumina HiSeq 2000	356	Selection comprised “218 previously reported causative genes and additional PID- related genes recently published or presented at scientific conferences at the time of the gene panel design, as well as several genes suspected to cause immunodeficiency”	98.31% at least 4 times	NR	NR	NR	15 genes were not well covered.
Abolhassani et al. (2018)	Array-based targeted PID panel + WES	Array-based targeted PID panel: PID v2 panels and Ion Torrent S5	Array-based targeted PID panel: 200	“..Sanger sequencing was performed on the most likely genes”.	Array-based targeted PID panel: Average coverage of 335 times	NR	NR	NR	NA
		WES: NR	WES: 365	“.. in whom Sanger failed or who had clinical presentation resembling several genetic defects, targeted NGS was performed..”	WES: Average on target coverage of 50 times				
Abolhassani et al. (2019)	WES	NR	NA^1^	NR	NR	NR	NR	NR	NA

*NR*, not reported; *M*, median; *a*, average

^1^Not applicable since the aim was also to discover new genetic defects

**Table 3:** Rationale for evaluation of variants and clinical performance of NGS-based tests

Authors	Diagnostic yield	Variants included for evaluation	Additional CNV analyses	Tools/methods for variant pathogenicity evaluation	Classification/reporting of variants	Clinical implications
Analysis of unsorted PIDs						
Nijman et al.	4/26 = 15%	Nonsense, missense, frameshift and variants in putative splice-site consensus sequences Comparison to in-house gene-specific mutation databases and HGMD database. Variants were relevant if present in < 5% of dbSNP/Exome variant server/1000 Genomes databases.	Yes	PolyPhen2, Sorting Intolerant From Tolerant (SIFT), genomic evolutionary rate profiling and Grantham scores, and the Alamut mutation interpretation software program.	Nonsense, frameshift, and canonical splice site variants were considered pathogenic. For remaining variants software tools were used.	All four patients were reclassified according to their mutation. 3 patients had atypical presentations.
Stoddard et al.	18/120 = 15%	Coding exons and highly conserved intronic regions for three genes were included. Other non-coding regions, including promoters and other regulatory regions were not included.	No	ANNOVAR.	NR	NR
Moens et al.	6/15 = 40%	Nonsense, missense, splice site-disrupting SNVs and INDEL variants predicted to disrupt a transcript’s reading frame. Heterozygous variants having an rs number were retained only if they were annotated as having clinical significance. Homozygous variants were retained irrespective of their annotation	Yes	For each patient, a list of potential disease causing mutations was prepared based on phenotypic filtering criteria.	Potential disease causing mutations that matched the phenotype.	NR
Al-Mousa et al.	35/139 = 25%	Nonsense, frameshift and canonical splice site mutations. Comparison to HGMD, 1000 Genomes and in-house databases. All variants with > 1% frequency were filtered out.	Yes	ANNOVAR, PolyPhen2, mutation taster.	Nonsense, frameshift and canonical splice site mutations were considered pathogenic. Remaining variants were included if identified as pathogenic by PolyPhen and mutation taster.	Several patients had previous negative findings despite extensive molecular studies. They represented atypical presentations of known PIDs.
Gallo et al.	7/45 = 16%	Coding and splice variants with a minor allele frequency of 1% or less in the CMH internal database, the Exome Variant Server or the Exome aggregation Consortium.	No	Symptom- and sign-assisted genome analysis (SSAGA), Phenomenizer data, SIFT and PolyPhen2. Functional assays: Homozygous or heterozygous variants already reported that were related to any immunological clinical phenotype; Variants in genes implicated in a molecular pathway related to the phenotype were considered if associated with any functional alteration, which was even partially consistent, with the clinical phenotype; All the genetic variants of genes that were probably unrelated to the molecular pathway suspected to be involved in the pathogenesis of the disease were excluded from the functional studies, but reported in an ad hoc repository.	Functional assays were performed to prove the impact of variants identified.	Possibly disease associated mutations in 15/45 = 33% other patients. Of the genetically diagnosed patients, 3 presented with atypical presentation.
Stray-Pedersen et al.	110/278 = 40%	Comparison of variants to Exome Sequencing Project, 1000 Genomes, the Exome aggregation Consortium and in-house databases. If no PID-causing variants were found in the selected genes, complete exome data was investigated. Variants were selected based on rarity and previously published cases with the same gene variants. Further analysis if variants were not present in the ExAC database if homozygous, or if frequency of < 0.0001 in the heterozygous/hemizygous state.	Yes	ANNOVAR Evaluation of possible genotype-phenotype correlation based on gene function, pathway, expression pattern, and results from model organisms. Computational prediction tools. PhyloP, GERP, SIFT, PolyPhen-2, LRT, and MutationTaster.	Classification of variants according to the American College of Medical Genetics and Genomics guidelines, inclusion of class 5, 4, and 3 but only if phenotype was consistent with genotype.	The clinical diagnosis of 55% of the genetically diagnosed patients was altered because of WES data. In 25% of patients, disease management was changed. In 1 patient, stem cell transplant may in hindsight not have been performed if the genetic diagnosis would have been known.
Rae et al.	13/27 = 46%	Nonsense, frameshift, missense, SNPs, INDELs and canonical splice site mutations. Further analysis if frequency of < 0.01 in the exome aggregation consortium.	No	PolyPhen, SIFT, and Ensembl variant effector predictor. Integrative Genomics Viewer.	Inclusion of variants that were pathogenic, likely pathogenic according to American College of Medical Genetics, or with clinical implications for PID disease management assessed through clinical risk modification of PIDs.	Genetic information had implications in management and treatment in 37% of the total cohort.
Bisgin et al.	17/37 = 46%	NR	No	Analysis for mutations were performed by SIFT, Polyphen-2 and MutationTaster	Causative mutations and novel mutations	It was suggested to include the diagnostic algorithm from this study for clinical use.
Analysis of specific PID disease categories						
Maffucci et al.	15/50 = 30%	Screening through the Human Gene Mutation Database. Heterozygous and homozygous mutations excluded if their allele frequency was > 0.01% and > 1.0% respectively in the Exome Aggregation Consortium Database.	No	Familial segregation analysis if samples were available. Analysis of confirmed mutations using computational predictors of mutation severity, including combined annotation-dependent depletion (CADD), and were compared with the gene-specific mutation significance cutoff (MSC).	Selected mutations were considered likely disease-causing.	Disease associated mutations in 8 other patients = 16%.
Yu et al.	14/20 = 70%	Nucleotide changes observed in more than 5% of aligned reads were called and reviewed. Deleterious mutations and novel variants were further analyzed.	Yes	NR	Mutations were considered disease-causing.	NR
Mukda et al.	12/25 = 50%	Non-synonymous SNPs and coding exons with the ability to alter amino acid sequence of a protein. Minor allele frequency in the hg19 reference genome was limited to < 5% or novel. Additional comparison of variants to an in-house database.	No	SIFT-score and PolyPhen. Gene expression analysis.	Included variant findings were categorized as pathogenic according to allele frequency, likely pathogenic, variant of unknown significance, and benign.	NR
Erman et al.	6/19 = 33%	Rare nonsense and missense variants within the exons as well as splice-site variants of the targeted genes. Variants with a frequency of < 0.01% were included	No	Variant effect prediction using SnpEff software	Disease-causing genetic defects based on the respective patients’ phenotype.	NR
Abolhassani et al. (2018)	189/243 = 79%	NR	Yes	NR	NR	NR
Abolhassani et al. (2019)	86/126 = 68%	Frequency of < 1% of in house database with more than 300 unrelated individuals sequenced, < 1% of 1111 unrelated individuals in the Greater Middle Eastern Database, < 0.01% of Exome Aggregation Consortium database, and < 0.01% of the genome AD database.	No	According to tools described in Fang et al. (2016), which are: CADD, PolyPhen2, GERP, GWAWA, and MutationTaster	Classification of variants according to the American College of Medical Genetics and Genomics criteria (inclusion of pathogenic or likely pathogenic variants). Study includes SNVs, missense, nonsense, splice-site, insertion/deletion, in-frame, frameshift and large deletion mutations.	In 26 patients (20.6%), therapy has switched from Ig replacement therapy to HSCT. In 15 patients (11.9%), regular screening for cancer was added to routine management with defects in their DNA repair system. Forty-nine patients (38.8%) were aided in family counseling, leading to prenatal diagnosis in 25 families (19.8%).

*NR*, not reported

The mean reading depth ranged from 80 to 1337. Five studies described the sensitivity of their technique and reported an overall sensitivity of 83–100%. Nijman et al. made a distinction between SNVs and indels [23], whereas Al-Mousa et al. analyzed SNVs and short indels on the one hand and CNVs on the other [24]. Both found higher sensitivity rates for SNVs, or SNVs and indels respectively. Specificity ranged from 45 to 99.9% with the lowest percentage found for the analysis of CNVs in the study by Al-Mousa et al.

### Clinical Performance

All studies explored the diagnostic yield of their NGS analyses, the results of which can be found in Table 3. The percentage of patients who were genetically diagnosed ranged from 15 to 79%. The diagnostic yield of NGS in mixed PID groups ranged from 15 to 46% (median = 25%). Within the specific PID subcategories, these values ranged from 30 to 79% (median = 42%), but were less evenly distributed. All studies described the pipeline used to establish pathogenicity of detected variants, except one. We evaluated information regarding several steps in the process. The results of variants on the amino acid sequence, such as missense-, nonsense-, or splice-site-altering, were collected. Other variables include the reference database to which the variants were compared, variant analyses, and further studies providing lines of evidence regarding the pathogenicity of a specific variant, such as parental cosegregation analysis, functional assays, and genotype-phenotype linkage. Cutoff values for comparison with healthy population databases differed between studies. Nijman et al. and Mukda et al. reported all variants present in < 5% of a healthy cohort [23, 25]. The other studies used a lower cutoff value of < 1%. Several studies mentioned the clinical impact of their analyses on patients. Stray-Pedersen et al. described significant changes in management in up to 25% of cases [26]. Rae et al. found an even higher number of 37% [17]. Four studies found a number of patients that were clinically reclassified according to their molecular diagnosis.

### Critical Appraisal

The results of the critical appraisal can be found in Appendix 2, Table 5. The reporting of variables was found to be incomplete for seven studies according to the aforementioned criteria. All articles explained the aim of their research, confirmed variants by Sanger sequencing, and proposed a set of possible implications of the research. The included studies scored relatively low on patient eligibility criteria, clinical background of their included patients, and analysis of sensitivity.

## Discussion

In this review, we analyzed test characteristics and performance of next generation sequencing techniques in patients with clinically defined, but genetically undiagnosed PIDs. After a systematic search, we collected fourteen studies describing the diagnostic yield of NGS in PID patients. A broad range in diagnostic yields was found (15–79%). This was explained by methodological differences (e.g., number of PID-related genes evaluated) and by different a priori risks for monogenetic causes of PID between the study populations. Overall, NGS-based evaluations performed well in a clinical setting.

Several studies described the clinical impact of their diagnoses. It was previously described that a genetic diagnosis is important for understanding the molecular mechanism of disease, for initiation of targeted therapy, for family counseling and reproductive advice, and because it can end the so called “diagnostic Odyssey” [22]. We found eight papers describing these clinical implications; they were frequent and ranged from changes in therapeutic approach to screening for malignancies [27].

A number of observations can be made regarding the patient populations in this review. First, as primary immunodeficiencies are rare disorders, there were few large cohorts describing specific PID phenotypes [2], with the exception of the studies by Abolhassani et al. reporting large cohorts in patients with PID subcategories [22, 27]. Stray-Pedersen et al. found varying diagnostic yields between disease subgroups in their cohort, ranging from 13% for autoinflammatory disorders to 100% for patients with SCID [26]. This illustrates that NGS may be more useful for certain PID sub-populations than others, depending on factors such as complexity of the underlying genetic mechanisms, parental consanguinity rate, and environmental factors.

Second, eight out of 14 studies were performed in Western countries, which could make the results less representative for other regions. As the prevalence of PIDs varies greatly due to differences in carrier rates of mutations, parental consanguinity rates, and varying patterns of expression, the descent of the included patients and the location where the research was performed may have significantly influenced results [28]. Especially the two studies by Abolhassani et al. illustrate this, reporting overall diagnostic yields of 79 and 68% in highly consanguineous patient populations. Even in a subcategory of patients with antibody deficiencies, a diagnostic yield of 68% was reported [22, 27].

Third, six studies mention that a subset of patients had received extensive genetic testing (for example, by Sanger sequencing) prior to NGS-based evaluation. This may have influenced the yield of NGS-based testing in a negative way as obvious well-known candidate mutations were likely to have been identified by prior Sanger-based testing. This difference is illustrated by Al-Mousa et al. where a higher diagnostic yield was found for new cases without any previous genetic work-up in comparison with cases without prior genetic evaluation [24].

Apart from the type of patients undergoing testing, the overall performance of NGS depends on several other technical variables, including sequencing method, number of genes analyzed, and interpretation pipelines. Due to the limited number of studies included in this review, statistical analyses to identify factors that accounted for differences in yield over time could not be performed. Most studies used array-based PID panels, and no clear difference in diagnostic yield was found between studies using this approach and those using WES. No studies using WGS were included in this review, although we identified one case series using WGS that identified the genetic cause of disease in 6/6 patients, indicating the diagnostic potential of WGS [29]. Regarding the role of the number of PID-related genes evaluated per study, we found that most studies included a set of genes referenced in the IUIS guidelines. A range of 12–365 PID-related genes in the panels for the specific PID populations was found and 60–571 in the panels for unsorted PID populations. The heterogeneity in the number of genes used in the different studies illustrates that currently only limited consensus exists as to which genes should be investigated in patients with suspected PIDs. Due to the frequent discovery of new PID-related genes, any standardized gene set in a PID panel will have to undergo regular updates to include new genes; this may be especially challenging for PIDs caused by a variety of genes such as CVID [30, 31]. In the Netherlands, in an attempt to provide uniform testing, all genetic laboratories have adopted the same nationwide PID gene panel which undergoes three monthly updates by consensus meetings.

Another technical factor that influences the diagnostic yield of NGS is coverage of nucleotides. Low coverage decreases the likelihood of NGS to retrieve pathogenic mutations, and may be caused by a variety of reasons including insufficient reads of a specific region and mapping problems. For example, Nijman et al. reported a set of nine genes that could not be adequately sequenced by NGS, an issue also reported in several other studies [23, 32, 33]. This may be due to the presence of pseudogenes (for example *IKBKG* and *NCF1*) or to high CG-content [34]. These regions cannot be reliably analyzed using a NGS approach based on short reads, and should be targeted using alternative techniques. Long read technologies with low SNV error rates will solve these limitations, but are still under development. The third reason for low coverage may be the presence of CNV’s, which may be missed by NGS and are identified more reliably by WGS-based techniques [4, 35]. The fact that CNV analyses can provide a considerable percentage of additional genetic diagnoses (on average 4.2% in the three studies that provided these data) indicates that CNV analyses can be a very valuable part of the diagnostic pipeline [26].

Finally, the clinical performance of NGS has been shown to depend on method of subsequent interpretation pipelines. We noted that different reference sets of DNA variants were used in the pathogenicity analysis between the studies. In the studies by Nijman et al. and Mukda et al., the cutoff values for frequency of SNPs in healthy control population was < 5% whereas most other studies used < 1% or even lower cutoff values [23, 25]. Moreover, most studies did not include intronic or synonymous variants in their analyses. Substantial heterogeneity was also found between papers in the means used to evaluate the pathogenicity of the DNA variants. Stray-Pedersen and Rae followed the American College of Medical Genetics and Genomics guidelines recommended for Mendelian disorders, while others gave limited description how they classified DNA variants [17, 26]. Software pathogenicity prediction tools were employed most often, sometimes in combination with functional assays, familial cosegregation analysis, or other additional analyses. As the final diagnostic yield greatly depends on the type and quality of these procedures, some variants may have been falsely marked as pathogenic, whereas other disease-causing variants may have been missed*.* The heterogeneity between the articles on this topic illustrates the need for more standardized procedures to evaluate the disease-causing potential of mutations.

The heterogeneity between the included studies is one of the most important limitations of this review. An average diagnostic yield is difficult to establish as a broad variety of factors has to be taken into account. For instance, we noted large differences in yields between the studies performed in populations with low consanguinity rates versus high consanguinity rates. A second limitation is that case studies and case series < *n* = 10 cases were excluded. Many of these reports did in fact identify causative genetic mutations in the majority of their patients. Pooling of these patients in future studies could provide additional information. Studies that included patients with VEO-IBD phenotypes were also excluded. Suzuki et al., for instance, used WES in order to identify the molecular mechanism of disease for pediatric IBD in 35 patients. Fifty-five genes were investigated, providing 14% of all patients with a genetic diagnosis [36]. Interestingly, all diagnosed patients were found to have PID-associated mutations. Future research could investigate the efficacy of targeted NGS PID-panels within this and other specific PID-related patient groups.

NGS is a relatively new technique, and has given rise to several questions outside the direct scope of this review. First, the more genes sequenced, the more variants will be detected of which the clinical significance is unknown. When misinterpreted, these variants of unknown significance (VUS) may hamper proper treatment and may cause unnecessary distress to patients. A second issue (especially in the application of unfiltered WES or WGS) is the possibility of discovering incidental findings: pathogenic mutations in genes related to other illnesses [17]. This is an important consideration as unfiltered WES and WGS are likely to become increasingly more relevant in the future [4]. Due to its comprehensive nature, WES and WGS without PID filter have the ability to provide patients with an alternative genetic diagnosis than PID. Careful counseling of patients on this topic is indispensable. Currently, however, the large amounts of data generated by WES and WGS complicate its use as a routine first-line investigation, reason why currently the output of WES and WGS is usually interpreted first with the application of a PID filter [34]. Third, even though NGS can be cheaper than Sanger sequencing in certain groups of patients, it remains expensive. Further research should focus on the most appropriate place to use NGS in the diagnostic pipeline in order to ensure the highest level of cost-effectiveness. Last, even though the costs of NGS are decreasing, the application of Sanger sequencing remains important in several situations. For example, in patients with a high suspicion of only one or two disease-related genes, Sanger sequencing can be more effective than NGS due to its high sensitivity and specificity and usually relatively short time to diagnosis. Finally, NGS may be a quicker and more comprehensive alternative, but it can fail to detect certain mutations. For this reason, Sanger sequencing is also required for the sequencing of parts of genes that are poorly covered by NGS. Sanger sequencing can also be used for confirmation of pathogenic mutations identified by NGS, but this is only necessary when results are inconclusive.

In conclusion, this systematic review shows that NGS has the ability to contribute significantly to the identification of molecular mechanisms in PID patients, thereby altering clinical management. This highlights the potential value of NGS in clinical practice. The diagnostic yields presented in this review highly depended on their context such as clinical background and technological performance of the diagnostic method. Therefore, further research should be performed in order to determine the efficacy and associated costs of NGS in patients with PIDs. Moreover, a more standardized means of analysis should be conceptualized in order to correctly identify the causative genetic defect in PID patients.

## Appendix 1

**Table 4:** Search string

**P**	PIDD OR PID OR primary immunodeficiency OR primary immune deficiency
**I**	NGS OR next generation sequenc* OR WES OR whole exome sequenc* OR targeted exome sequenc* OR WGS OR whole genome sequenc*
**C**	/
**O**	/

Pubmed: P AND I = 196 results

Embase: P AND I = 297 results

## Appendix 2

**Table 5:** Critical appraisal based on the STARD 2015 criteria

Main author	Introduction	Methods						Results				Discussion	
	Aim article explained?	Description of source selected patients?	Description of patient eligibility criteria?	Mixed phenotype studies: inclusion of diverse population?	Detailed rationale for included genes?	Detailed description for evaluation of significance of variant?	Description of test protocol	Description of clinical background of all patients?	Description of clinical background of diagnosed patients?	Evaluation of sensitivity?	Confirmation of variants by Sanger?	Description of study limitations?	Description of implications?
Nijman	Yes	Yes	No	No	Yes	Yes	Yes	No	Yes	Yes	Yes	Yes	Yes
Stoddard	Yes	No	No	Unclear	Yes	No	Yes	No	No	Yes	Yes	Yes	Yes
Moens	Yes	Yes	No	No	Yes	Yes	Yes	No	Yes	Yes	Yes	Yes	Yes
Al-Mousa	Yes	No	No	Yes	Yes	Yes	Yes	Yes	Yes	Yes	Yes	Yes	Yes
Maffucci	Yes	Yes	Yes	NA	No	Yes	Yes	No	Yes	No	Yes	No	Yes
Yu	Yes	No	No	NA	No		Yes	No	Yes	No	Yes	No	Yes
Gallo	Yes	No	Yes	Yes	Yes	Yes	Yes	No	Yes	Yes	Yes	Yes	Yes
Stray-Pedersen	Yes	Yes	Yes	Yes	Yes	Yes	Yes	No	Yes	No	Yes	Yes	Yes
Mukda	Yes	Yes	Yes	NA	No	Yes	Yes	Yes	Yes	No	Yes	Yes	Yes
Rae	Yes	Yes	Yes	Yes	No	Yes	Yes	No	No	No	Yes	Yes	Yes
Erman	Yes	Yes	No	NA	No	Yes	Yes	Yes	Yes	No	Yes	Yes	Yes
Abolhassani 2018	Yes	Yes	Yes	NA	No	No	Yes	Yes	Yes	No	No	No	Yes
Bisgin	Yes	Yes	No	Yes	No	Yes	Yes	No	Yes	No	Yes	Yes	No
Abolhassani 2019	Yes	Yes	Yes	NA	No	Yes	Yes	Yes	Yes	No	No	No	Yes

*NA*, not applicable

## Abbreviations

ALPS: autoimmune lymphoproliferative syndrome
BTK: Bruton’s tyrosine kinase
CID: Combined immunodeficiency
CMC: Chronic mucocutaneous candidiasis
CNV: Copy number variant
CVID: Common variable immunodeficiency
GC: Guanine-cytosine
HLH: Hemophagocytic lymphohistiocytosis
(VEO)-IBD: (Very early-onset) inflammatory bowel disease
MHC: Major histocompatibility complex
NGS: Next generation sequencing
PID: Primary immunodeficiency
SCID: Severe combined immunodeficiency
SNP: Single-nucleotide polymorphism
SNV: Single-nucleotide variant
VUS: Variant of unknown significance
WES: Whole exome sequencing
WGS: Whole genome sequencing
XLP: X-linked lymphoproliferative disease

## References

[1] van der Spek J, Groenwold RHH, van der Burg M, van Montfrans JM (2015). TREC based newborn screening for severe combined immunodeficiency disease: a systematic review. J Clin Immunol.

[2] Gray PEA, Namasivayam M, Ziegler JB (2012). Recurrent infection in children: when and how to investigate for primary immunodeficiency?. J Paediatr Child Health.

[3] Shillitoe B, Bangs C, Guzman D, Gennery AR, Longhurst HJ, Slatter M, Edgar DM, Thomas M, Worth A, Huissoon A, Arkwright PD, Jolles S, Bourne H, Alachkar H, Savic S, Kumararatne DS, Patel S, Baxendale H, Noorani S, Yong PFK, Waruiru C, Pavaladurai V, Kelleher P, Herriot R, Bernatonienne J, Bhole M, Steele C, Hayman G, Richter A, Gompels M, Chopra C, Garcez T, Buckland M (2018). The United Kingdom primary immune deficiency (UKPID) registry 2012 to 2017. Clin Exp Immunol.

[4] Heimall JR, Hagin D, Hajjar J, Henrickson SE, Hernandez-Trujillo HS, Tan Y, Kobrynski L, Paris K, Torgerson TR, Verbsky JW, Wasserman RL, Hsieh EWY, Blessing JJ, Chou JS, Lawrence MG, Marsh RA, Rosenzweig SD, Orange JS, Abraham RS (2018). Use of genetic testing for primary immunodeficiency patients. J Clin Immunol.

[5] Gallo V, Dotta L, Giardino G, Cirillo E, Lougaris V, D’Assante R, et al. Diagnostics of primary immunodeficiencies through next-generation sequencing. Front Immunol. 2016;7. 10.3389/fimmu.2016.00466. 10.3389/fimmu.2016.00466 PMC5098274 27872624

[6] Oliveira JB, Fleisher TA. Laboratory evaluation of primary immunodeficiencies. J Allergy Clin Immunol. 2010;7. 10.3389/fimmu.2016.00466. 10.1016/j.jaci.2009.08.043 PMC3412511 20042230

[7] Alkan G, Keles S, Reisli İ (2018). Evaluation of clinical and immunological characteristics of children with common variable immunodeficiency. Int J Pediatr.

[8] Stoddard JL, Niemela JE, Fleisher TA, Rosenzweig SD. Targeted NGS: a cost-effective approach to molecular diagnosis of PIDs. Front Immunol. 2014;5. 10.3389/fimmu.2014.00531. 10.3389/fimmu.2014.00531 PMC4217515 25404929

[9] Seleman M, Hoyos-Bachiloglu R, Geha RS, Chou J. Uses of next-generation sequencing technologies for the diagnosis of primary Immunodeficiencies. Front Immunol. 2017;8. 10.3389/fimmu.2017.00847. 10.3389/fimmu.2017.00847 PMC5522848 28791010

[10] Notarangelo LD, Sorensen R (2008). Is it necessary to identify molecular defects in primary immunodeficiency disease?. J Allergy Clin Immunol.

[11] Raje N, Soden S, Swanson D, Ciaccio CE, Kingsmore SF, Dinwiddie DL (2014). Utility of next generation sequencing in clinical primary immunodeficiencies. Curr Allergy Asthma Rep.

[12] Bisgin A, Boga I, Yilmaz M, Bingol G, Altintas D (2018). The utility of next-generation sequencing for primary immunodeficiency disorders: experience from a clinical diagnostic laboratory. Biomed Res Int.

[13] Picard C, Bobby Gaspar H, Al-Herz W, Bousfiha A, Casanova JL, Chatila T, Crow YJ, Cunningham-Rundles C, Etzioni A, Franco JL, Holland SM, Klein C, Morio T, Ochs HD, Oksenhendler E, Puck J, Tang MLK, Tangye SG, Torgerson TR, Sullivan KE (2018). International Union of Immunological Societies: 2017 primary immunodeficiency diseases committee report on inborn errors of immunity. J Clin Immunol.

[14] Picard C, Fischer A (2014). Contribution of high-throughput DNA sequencing to the study of primary immunodeficiencies. Eur J Immunol.

[15] Engelhardt KR, Xu Y, Grainger A, Germani Batacchi MGC, Swan DJ, Willet JDP, Abd Hamid IJ, Agyeman P, Barge D, Bibi S, Jenkins L, Flood TJ, Abinun M, Slatter MA, Gennery AR, Cant AJ, Santibanez Koref M, Gilmour K, Hambleton S (2017). Identification of heterozygous single- and multi-exon deletions in IL7R by whole exome sequencing. J Clin Immunol.

[16] van Schouwenburg PA, Davenport EE, Kienzler A-K, Marwah I, Wright B, Lucas M, Malinauskas T, Martin HC, Lockstone HE, Cazier JB, Chapel HM, Knight JC, Patel SY, WGS500 Consortium (2015). Application of whole genome and RNA sequencing to investigate the genomic landscape of common variable immunodeficiency disorders. Clin Immunol.

[17] Rae W, Ward D, Mattocks C, Pengelly RJ, Eren E, Patel SV, Faust SN, Hunt D, Williams AP (2018). Clinical efficacy of a next-generation sequencing gene panel for primary immunodeficiency diagnostics. Clin Genet.

[18] Fang M, Abolhassani H, Lim CK, Zhang J, Hammarström L (2016). Next generation sequencing data analysis in primary immunodeficiency disorders - future directions. J Clin Immunol.

[19] Bossuyt PM, Reitsma JB, Bruns DE, Gatsonis CA, Glasziou PP, Irwig LM, et al. Towards complete and accurate reporting of studies of diagnostic accuracy: the STARD initiative. standards for reporting of diagnostic accuracy. Clin Chem. 2003 Jan;49(1):1–6. Available from http://www.ncbi.nlm.nih.gov/pubmed/12507953. 10.1373/49.1.1 12507953

[20] Yu H, Zhang VW, Stray-Pedersen A, Hanson IC, Forbes LR, de la Morena MT, Chinn IK, Gorman E, Mendelsohn NJ, Pozos T, Wiszniewski W, Nicholas SK, Yates AB, Moore LE, Berge KE, Sorte H, Bayer DK, ALZahrani D, Geha RS, Feng Y, Wang G, Orange JS, Lupski JR, Wang J, Wong LJ (2016). Rapid molecular diagnostics of severe primary immunodeficiency determined by using targeted next-generation sequencing. J Allergy Clin Immunol.

[21] Maffucci P, Filion CA, Boisson B, Itan Y, Shang L, Casanova J-L, et al. Genetic diagnosis using whole exome sequencing in common variable immunodeficiency. Front Immunol. 2016;7. 10.3389/fimmu.2016.00220. 10.3389/fimmu.2016.00220 PMC4903998 27379089

[22] Abolhassani H, Aghamohammadi A, Fang M, Rezaei N, Jiang C, Liu X, et al. Clinical implications of systematic phenotyping and exome sequencing in patients with primary antibody deficiency. Genet Med [Internet. 2019;21(1):243–51 Available from: http://www.ncbi.nlm.nih.gov/pubmed/29921932. 10.1038/s41436-018-0012-x 29921932

[23] Nijman IJ, van Montfrans JM, Hoogstraat M, Boes ML, van de Corput L, Renner ED, van Zon P, van Lieshout S, Elferink MG, van der Burg M, Vermont CL, van der Zwaag B, Janson E, Cuppen E, Ploos van Amstel JK, van Gijn ME (2014). Targeted next-generation sequencing: a novel diagnostic tool for primary immunodeficiencies. J Allergy Clin Immunol.

[24] Al-Mousa H, Abouelhoda M, Monies DM, Al-Tassan N, Al-Ghonaium A, Al-Saud B (2016). Unbiased targeted next-generation sequencing molecular approach for primary immunodeficiency diseases. J Allergy Clin Immunol.

[25] Mukda E, Trachoo O, Pasomsub E, Tiyasirichokchai R, Iemwimangsa N, Sosothikul D, Chantratita W, Pakakasama S (2017). Exome sequencing for simultaneous mutation screening in children with hemophagocytic lymphohistiocytosis. Int J Hematol.

[26] Stray-Pedersen A, Sorte HS, Samarakoon P, Gambin T, Chinn IK, Coban Akdemir ZH, Erichsen HC, Forbes LR, Gu S, Yuan B, Jhangiani SN, Muzny DM, Rødningen OK, Sheng Y, Nicholas SK, Noroski LM, Seeborg FO, Davis CM, Canter DL, Mace EM, Vece TJ, Allen CE, Abhyankar HA, Boone PM, Beck CR, Wiszniewski W, Fevang B, Aukrust P, Tjønnfjord GE, Gedde-Dahl T, Hjorth-Hansen H, Dybedal I, Nordøy I, Jørgensen SF, Abrahamsen TG, Øverland T, Bechensteen AG, Skogen V, Osnes LTN, Kulseth MA, Prescott TE, Rustad CF, Heimdal KR, Belmont JW, Rider NL, Chinen J, Cao TN, Smith EA, Caldirola MS, Bezrodnik L, Lugo Reyes SO, Espinosa Rosales FJ, Guerrero-Cursaru ND, Pedroza LA, Poli CM, Franco JL, Trujillo Vargas CM, Aldave Becerra JC, Wright N, Issekutz TB, Issekutz AC, Abbott J, Caldwell JW, Bayer DK, Chan AY, Aiuti A, Cancrini C, Holmberg E, West C, Burstedt M, Karaca E, Yesil G, Artac H, Bayram Y, Atik MM, Eldomery MK, Ehlayel MS, Jolles S, Flatø B, Bertuch AA, Hanson IC, Zhang VW, Wong LJ, Hu J, Walkiewicz M, Yang Y, Eng CM, Boerwinkle E, Gibbs RA, Shearer WT, Lyle R, Orange JS, Lupski JR (2017). Primary immunodeficiency diseases: genomic approaches delineate heterogeneous Mendelian disorders. J Allergy Clin Immunol.

[27] Abolhassani H, Chou J, Bainter W, Platt CD, Tavassoli M, Momen T, Tavakol M, Eslamian MH, Gharagozlou M, Movahedi M, Ghadami M, Hamidieh AA, Azizi G, Yazdani R, Afarideh M, Ghajar A, Havaei A, Chavoshzadeh Z, Mahdaviani SA, Cheraghi T, Behniafard N, Amin R, Aleyasin S, Faridhosseini R, Jabbari-Azad F, Nabavi M, Bemanian MH, Arshi S, Molatefi R, Sherkat R, Mansouri M, Mesdaghi M, Babaie D, Mohammadzadeh I, Ghaffari J, Shafiei A, Kalantari N, Ahanchian H, Khoshkhui M, Soheili H, Dabbaghzadeh A, Shirkani A, Nasiri Kalmarzi R, Mortazavi SH, Tafaroji J, Khalili A, Mohammadi J, Negahdari B, Joghataei MT, al-Ramadi BK, Picard C, Parvaneh N, Rezaei N, Chatila TA, Massaad MJ, Keles S, Hammarström L, Geha RS, Aghamohammadi A (2018). Clinical, immunologic, and genetic spectrum of 696 patients with combined immunodeficiency. J Allergy Clin Immunol.

[28] Al-Herz W, Aldhekri H, Barbouche M-R, Rezaei N (2014). Consanguinity and primary immunodeficiencies. Hum Hered.

[29] Mousallem T, Urban TJ, McSweeney KM, Kleinstein SE, Zhu M, Adeli M (2015). Clinical application of whole-genome sequencing in patients with primary immunodeficiency. J Allergy Clin Immunol.

[30] Kienzler A-K, Hargreaves CE, Patel SY (2017). The role of genomics in common variable immunodeficiency disorders. Clin Exp Immunol.

[31] Ameratunga R, Lehnert K, Woon S-T, Gillis D, Bryant VL, Slade CA, Steele R (2018). Review: diagnosing common variable immunodeficiency disorder in the era of genome sequencing. Clin Rev Allergy Immunol.

[32] Moens LN, Falk-Sörqvist E, Asplund AC, Bernatowska E, Smith CIE, Nilsson M (2014). Diagnostics of primary immunodeficiency diseases: a sequencing capture approach. PLoS One.

[33] Erman B, Bilic I, Hirschmugl T, Salzer E, Boztug H, Sanal Ö, Çağdaş Ayvaz D, Tezcan I, Boztug K (2017). Investigation of genetic defects in severe combined immunodeficiency patients from Turkey by targeted sequencing. Scand J Immunol.

[34] Meyts I, Bosch B, Bolze A, Boisson B, Itan Y, Belkadi A, Pedergnana V, Moens L, Picard C, Cobat A, Bossuyt X, Abel L, Casanova JL (2016). Exome and genome sequencing for inborn errors of immunity. J Allergy Clin Immunol.

[35] Whitford W, Lehnert K, Snell RG, Jacobsen JC (2019). Evaluation of the performance of copy number variant prediction tools for the detection of deletions from whole genome sequencing data. J Biomed Inform.

[36] Suzuki T, Sasahara Y, Kikuchi A, Kakuta H, Kashiwabara T, Ishige T, Nakayama Y, Tanaka M, Hoshino A, Kanegane H, Abukawa D, Kure S (2017). Targeted sequencing and immunological analysis reveal the involvement of primary immunodeficiency genes in pediatric IBD: a Japanese multicenter study. J Clin Immunol.

